# The effects of probiotic supplementation on metabolic status in type 2 diabetic patients with coronary heart disease

**DOI:** 10.1186/s13098-018-0353-2

**Published:** 2018-06-19

**Authors:** Fariba Raygan, Zohreh Rezavandi, Fereshteh Bahmani, Vahidreza Ostadmohammadi, Mohammad Ali Mansournia, Maryam Tajabadi-Ebrahimi, Shokoofeh Borzabadi, Zatollah Asemi

**Affiliations:** 1 Department of Cardiology, School of Medicine, Kashan University of Medical Sciences, Kashan, Iran; 2 Research Center for Biochemistry and Nutrition in Metabolic Diseases, Kashan University of Medical Sciences, Kashan, Iran; 3 Department of Epidemiology and Biostatistics, School of Public Health, Tehran University of Medical Sciences, Tehran, Iran; 4 Science Faculty, Islamic Azad University, Central Branch, Tehran, Iran; 5 Department of Biology, Science and Research Branch, Islamic Azad University, Tehran, Iran

**Keywords:** Probiotic, Coronary heart disease, Metabolic status, Type 2 diabetes mellitus

## Abstract

**Background:**

This study was conducted to evaluate the effects of probiotic supplementation on metabolic profiles in diabetic patients with coronary heart disease (CHD).

**Methods:**

This randomized, double-blind, placebo-controlled trial was performed among 60 diabetic patients with CHD, aged 40–85 years at a cardiology clinic in Kashan, Iran, from October 2017 through January 2018. Patients were randomly divided into two groups to take either probiotic supplements (n = 30) or placebo (n = 30) for 12 weeks. Fasting blood samples were taken at the beginning of the study and after the 12-week intervention to determine related markers.

**Results:**

After 12-week intervention, probiotic supplementation significantly decreased fasting plasma glucose (β − 20.02 mg/dL; 95% CI − 33.86, − 6.17; *P *= 0.005), insulin (β − 2.09 µIU/mL; 95% CI − 3.77, − 0.41; *P *= 0.01), insulin resistance (β − 0.50; 95% CI − 0.96, − 0.03; *P *= 0.03) and total-/HDL-cholesterol ratio (β − 0.27; 95% CI − 0.52, − 0.03; *P *= 0.02), and significantly increased insulin sensitivity (β 0.008; 95% CI 0.001, 0.01; *P *= 0.02) and HDL-cholesterol levels (β 2.52 mg/dL; 95% CI 0.04, 5.00; *P *= 0.04) compared with the placebo. Moreover, probiotic supplementation led to a significant reduction in serum high sensitivity C-reactive protein (β − 0.88 mg/L; 95% CI − 1.39, − 0.38; *P *= 0.001), and a significant elevation in total antioxidant capacity (β 108.44 mmol/L; 95% CI 47.61, 169.27; *P *= 0.001) and total glutathione levels (β 45.15 µmol/L; 95% CI 5.82, 84.47; *P *= 0.02) compared with the placebo. Probiotic supplementation did not affect other metabolic profiles.

**Conclusions:**

Overall, we found that probiotic supplementation for 12 weeks had beneficial effects on glycemic control, HDL-cholesterol, total-/HDL-cholesterol ratio, biomarkers of inflammation and oxidative stress in diabetic patients with CHD.

*Trial registration* Clinical trial registration number http://www.irct.ir: IRCT2017082733941N5

## Background

Type 2 diabetes mellitus (T2DM) is considered as a developing epidemic that influences adult population [1]. Urbanization, changing dietary pattern and inactive lifestyle contribute to an elevating incidence of T2DM and coronary heart disease (CHD) [2]. Hyperglycemia and insulin resistance in patients with T2DM exert considerable impacts on blood vessels, and demonstrated a capacity to increase the occurrence of retinopathy, nephropathy, and CHD [3]. Overall, patients with T2DM have higher risk of having cardiovascular disease (CVD) [4], which accounting for almost 60% of mortality related to diabetes [5].

The effects of probiotic supplementation, containing lactobacillus and bifidobacteria, on metabolic profiles have been reported frequently in previous studies in different populations, however to our best knowledge, probiotic supplementation have not been evaluated in diabetic patients with CHD. In addition, current evidence is inconclusive. In a study, oral administration of 2.5 × 10^9^ CFU/g probiotic containing *Bifidobacterium bifidum*, *Bifidobacterium lactis*, *Lactobacillus acidophilus*, *Lactobacillus brevis*, *Lactobacillus casei*, *Lactobacillus salivarius*, *Lactococcus lactis* and *Lactococcus lactis* twice a day for 12 weeks to patients with T2DM decreased insulin resistance [6]. Furthermore, in a meta-analysis conducted by Taylor et al. [7]. 6–8 weeks probiotic supplementation decreased insulin resistance in women with gestational diabetes (GDM), while did not affect lipid profiles and fasting glucose. Earlier, we have documented that co-supplementation of 8 × 10^9^ CFU/g probiotic, containing *Lactobacillus acidophilus, Bifidobacterium bifidum, Lactobacillus reuteri,* and *Lactobacillus fermentum* (each 2 × 10^9^) and 50,000 IU vitamin D every 2 weeks for 12 weeks to T2DM people with CHD had beneficial effects on mental health parameters, inflammatory markers, total antioxidant capacity, glycemic control and HDL-cholesterol, although there was no effect on other parameters of metabolic profiles [8]. In another investigation, taking probiotic, containing 112.5 × 10^9^ CFU/capsule of eight strains of lactic acid bacteria, for 8 weeks by women with GDM significantly modulated inflammatory markers, insulin and insulin resistance, although did not affect fasting glucose and HbA1c [9]. Discrepancies in these findings may be due to differences in study design, characteristics of study populations, dosage of probiotic and synbiotic used, type of bacteria used, and the duration of the intervention.

The beneficial effects of probiotic supplements on insulin resistance, lipid profiles, biomarkers of inflammation and oxidative damage may be explained through its impacts on scavenging superoxide and hydroxyl radicals [10], decreased inflammatory signaling [11] and weight reduction [12]. According to existing evidence that probiotic supplementation might have antioxidant and anti-inflammatory properties and glucose-lowering effects; we assumed that probiotic consumption may benefit diabetic patients with CHD. Therefore, this study was conducted to determine the effects of probiotic supplementation on metabolic status in diabetic patients with CHD.

## Methods

### Study population

This study was a randomized, double-blind, placebo-controlled trial registered in the Iranian registry of clinical trials (http://www.irct.ir: IRCT2017082733941N5) which performed at a cardiology clinic affiliated to Kashan University of Medical Sciences (KAUMS), Kashan, Iran, from October 2017 through January 2018. Inclusion criteria were as follows: patients with T2DM, aged 40–85 years old with 2- and 3-vessel CHD. T2DM was diagnosed based on the criteria of the American Diabetes Association [13]. Furthermore, the diagnosis of CHD was conducted following the American Heart Association guideline [14]. Exclusion criteria included consuming probiotic and/or synbiotic within 3 months prior to the intervention, taking prebiotic, antioxidant and/or anti-inflammatory supplements such as vitamin E, vitamin C and omega-3 fatty acids, taking antibiotics, having an acute myocardial infarction, a cardiac surgery in the past 3 months, renal or hepatic failure.

### Ethical statement

The research was done following the Declaration of Helsinki principals. The protocol of this study was approved by Research Ethics Committee, KAUMS, and Iran. Written informed consent was taken from all participants.

### Study design

Initially, we conducted a stratified randomization for all participants according to age, BMI, gender, the dosage and kind of medications, in order to decrease potential confounding effects. Then, participants in each stratum were randomly allocated into two treatment groups to take either probiotic supplements including *Bifidobacterium bifidum* 2 × 10^9^, *Lactobacillus casei* 2 × 10^9^, *Lactobacillus acidophilus* 2 × 10^9^ CFU/day (n = 30) or placebo (n = 30) for 12 weeks. Color, shape, size, and package of placebos and probiotics capsules were identical and made by Tak Gen Zist Pharmaceutical Company (Tehran, Iran). Randomization was conducted using computer-generated random numbers. Randomization and allocation were concealed from the investigators and participants until the final analyses were completed. The randomized allocation sequence, enrolling participants and allocating them into intervention groups were performed by a trained staff at cardiology clinic. At the beginning of the study, patients were requested to maintain their regular diet and levels of physical activity throughout the period of the trial. Compliance rate regarding the consumption of placebos and probiotic supplements was determined by examining the returned capsule containers. All patients completed 3-day dietary records at baseline, week 1, 4, 8 and 12 of treatment. To determine nutrient intakes of participants according to 3-day food records, we used Nutritionist IV software (First Databank, San Bruno, CA). Physical activity was quantified as metabolic equivalents (METs) in hours per day [15]. Anthropometric measures (Seca, Hamburg, Germany) were measured at baseline and end of the intervention at the cardiology clinic by a trained staff. Nutritionist also was blinded to the randomization assignments.

### Outcomes

Insulin metabolism was considered as primary outcome, however lipid profiles, biomarkers of inflammation and oxidative stress were considered as secondary outcomes. Fasting blood (10 mL) was taken at baseline and after the 12-week intervention at Kashan reference laboratory, Kashan, Iran. Insulin levels were measured using an ELISA kit (DiaMetra, Milano, Italy) with inter-assay and intra-assay coefficient variances (CVs) of lower than 5%. The homeostasis model of assessment-insulin resistance (HOMA-IR) and the quantitative insulin sensitivity check index (QUICKI) were calculated according to the standard formulas [16]. Enzymatic kits (Pars Azmun, Tehran, Iran) were used to determine fasting plasma glucose (FPG) and lipid profiles with inter- and intra-assay CVs of lower than 5%. Hs-CRP levels were measured using an ELISA kit (LDN, Nordhorn, Germany) with inter- and intra-assay CVs of lower than 7%. Nitric oxide (NO) was measured by Griess assay [17], total antioxidant capacity (TAC) the method reported by Benzie and Strain [18], total glutathione (GSH) by Beutler method [19], and MDA concentrations by the spectrophotometric test with inter- and intra-assay CVs of lower than 5% [20]. Systolic (SBP) and diastolic blood pressure (DBP) was measured using a sphygmomanometer (ALPK2, Zhejiang, China). Blood pressure values were reported in millimeters of mercury (mmHg).

### Statistical methods and sample size

We calculated sample size using the formula suggested for randomized clinical. Type one (α) and type two errors (β) were defined as 0.05, and 0.20 (power = 80%), respectively. According to the previous trial [21], we used 1.61 as the SD and 2.30 as the mean change (d) of HOMA-IR. Based on the formula, 25 participants were required in each group; after allowing for 5 dropouts in each group, the final sample size was 30 participants per intervention group.

The Kolmogorov–Smirnov test was conducted to determine the normal distribution of variables. The analyses were repeated using intention-to-treat (ITT) protocol. Independent-samples *t*-test was used to detect the differences in anthropometric measures and dietary intakes between two groups. Multiple linear regression models were applied to evaluate treatment impacts on study outcomes after adjusting for confounding parameters including; age, and BMI. The effect sizes were presented as the mean differences with 95% confidence intervals. Bootstrapping was also used as a sensitivity analysis of confidence intervals. Pearson Chi square test was applied for the comparison of categorical variables. P values < 0.05 were considered significant. The Statistical Package for Social Science version 18 (SPSS Inc., Chicago, Illinois, USA) was used for statistical analyses of this trial.

## Results

**Fig. 1:**
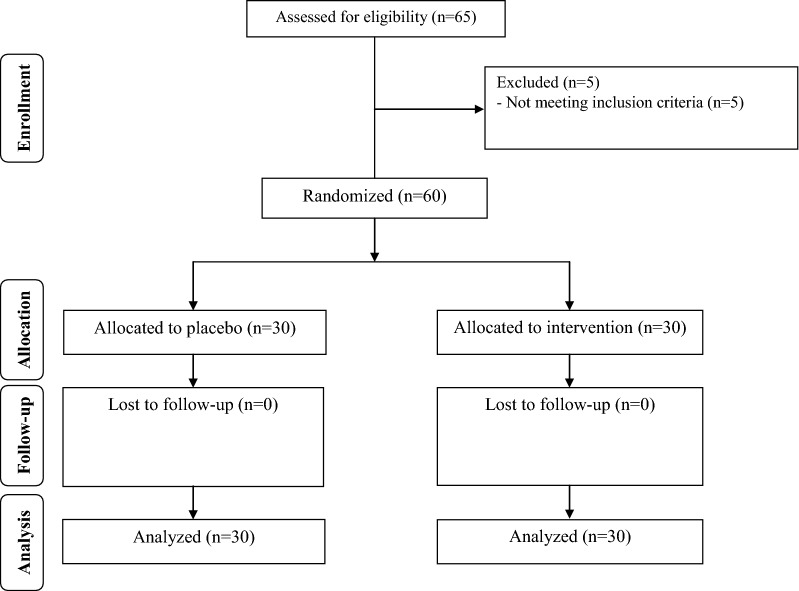
Summary of patient flow diagram

Sixty patients [probiotic (n = 30) and placebo (n = 30)] completed the trial (Fig. 1). In our study, the compliance rate was high, such that more than 90% of capsules were consumed throughout the study in both groups. No adverse effects were recorded in diabetic patients with CHD following probiotic supplementation.

There were no significant differences between two groups in terms of mean age, height, baseline weight, BMI, and mean changes in weight and BMI throughout the trial (Table 1). In addition, smoking status, taking antidiabetic and antilipidemic agents, incidence of hypertension, consumption of angiotensin converting enzymes inhibitors (ACEI), aldosterone receptor blockers (ARB) drugs and blocker drugs (β-blocker and calcium channel blocker) were not statistically different between two intervention groups.

**Table 1:** General characteristics of study participants at baseline study

	Placebo group (n = 30)	Probiotic group (n = 30)	*P* ^1^
Age (year)	61.8 ± 9.8	60.7 ± 9.4	0.64
Familial history (%)	10 (33.7)	11 (36.7)	0.78^†^
Height (cm)	163.2 ± 7.1	162.5 ± 7.2	0.72
Weight at study baseline (kg)	78.2 ± 11.8	80.2 ± 15.3	0.57
Weight at end-of-trial (kg)	78.2 ± 12.0	80.2 ± 15.3	0.56
Weight change (kg)	− 0.03 ± 1.1	0.04 ± 1.0	0.78
BMI at study baseline (kg/m^2^)	29.3 ± 4.1	30.3 ± 5.2	0.42
BMI at end-of-trial (kg/m^2^)	29.3 ± 4.1	30.3 ± 5.2	0.41
BMI change (kg/m^2^)	− 0.01 ± 0.4	0.06 ± 0.4	0.81
Smoking (%)	3 (10.0)	3 (10.0)	1.00^†^
Aspirin 80 mg (%)	30 (100)	30 (100)	1.00^†^
Statin (%)	30 (100)	30 (100)	1.00^†^
Insulin therapy (%)	8 (26.7)	7 (23.3)	0.76^†^
Antidiabetic drugs (%)			
Monotherpy	16 (72.7)	17 (70.8)	
Combination therapy	6 (27.3)	7 (29.2)	0.88^†^
Hypertension (%)	22 (73.3)	23 (76.7)	0.76^†^
ACEI/ARB drugs (%)	30 (100)	30 (100)	1.00^†^
Blocker drugs (%)			
β-blocker	28 (93.3)	29 (96.7)	
Calcium channel blocker	2 (6.7)	1 (3.3)	0.55^†^
Duration of DM (year)	6.8 ± 2.2	6.6 ± 1.9	0.61
Duration of CHD (year)	9.5 ± 2.2	9.3 ± 1.6	0.78

Data are means ± SDs

*ACEI* Angiontensin converting enzymes inhibitors, *ARB* Aldosterone receptor blockers, *CHD* coronary heart disease, *DM* diabetes mellitus

^1^Obtained from independent *t*-test

^†^Obtained from Pearson Chi square test

Based on 3-day dietary records, obtained at baseline and throughout the intervention, we observed no significant difference in macro- and micronutrients intake between the two groups (Table 2).

**Table 2:** Mean dietary intakes of study participants at study baseline and throughout the study

	Placebo group (n = 30)	Probiotic group (n = 30)	*P* ^1^
Energy (kcal/day)	2182 ± 215	2194 ± 224	0.83
Carbohydrates (g/day)	292.1 ± 38.1	298.5 ± 43.5	0.54
Protein (g/day)	82.5 ± 23.5	81.5 ± 17.8	0.85
Fat (g/day)	79.8 ± 14.6	79.0 ± 13.1	0.80
SFA (g/day)	26.1 ± 5.9	25.9 ± 5.5	0.92
MUFA (g/day)	22.7 ± 6.3	22.1 ± 5.7	0.69
PUFA (g/day)	23.4 ± 4.3	22.7 ± 4.3	0.50
Cholesterol (mg/day)	219.5 ± 128.9	205.1 ± 100.6	0.63
TDF (g/day)	19.1 ± 3.8	19.5 ± 4.1	0.69

Values are means ± SDs

*MUFAs* monounsaturated fatty acids, *PUFAs* polyunsaturated fatty acids, *SFAs* saturated fatty acids *TDF* total dietary fiber

^1^Obtained from independent samples *t*-test

Probiotic supplementation significantly decreased FPG (β − 20.02 mg/dL; 95% CI − 33.86, − 6.17; *P *= 0.005), serum insulin levels (β − 2.09 µIU/mL; 95% CI − 3.77, − 0.41; *P *= 0.01), HOMA-IR (β − 0.50; 95% CI − 0.96, − 0.03; *P *= 0.03) and total-/HDL-cholesterol ratio (β − 0.27; 95% CI − 0.52, − 0.03; *P *= 0.02), and significantly increased QUICKI (β 0.008; 95% CI 0.001, 0.01; *P *= 0.02) and HDL-cholesterol levels (β 2.52 mg/dL; 95% CI 0.04, 5.00; *P *= 0.04) compared with the placebo **(**Table 3). In addition, probiotic supplementation led to a significant reduction in serum hs-CRP (β − 0.88 mg/L; 95% CI − 1.39, − 0.38; *P *= 0.001), and a significant increase in plasma TAC (β 108.44 mmol/L; 95% CI 47.61, 169.27; *P *= 0.001) and GSH levels (β 45.15 µmol/L; 95% CI 5.82, 84.47; *P *= 0.02) compared with the placebo. Probiotic supplementation did not affect other metabolic profiles. When we adjusted the analysis for smoking status and familial history of CHD, findings remained intact.

**Table 3:** The effect of probiotic supplementation on metabolic status in type 2 diabetic patients with coronary heart disease

Variables	Placebo group (n = 30)		Probiotic group (n = 30)		Difference in outcome measures between probiotic and placebo treatment groups^a^	
	Baseline	Week 12	Baseline	Week 12	β (95% CI)	*P* ^2^
FPG (mg/dL)	128.8 ± 47.2	138.2 ± 33.5	133.8 ± 43.6	120.6 ± 38.7	− 20.02 (− 33.86, 6.17)	0.005
Insulin (μIU/mL)	13.8 ± 8.6	14.7 ± 8.5	14.3 ± 5.6	13.1 ± 5.2	− 2.09 (− 3.77, − 0.41)	0.01
HOMA-IR	4.5 ± 3.6	4.6 ± 3.2	4.8 ± 2.7	4.4 ± 2.4	− 0.50 (− 0.96, − 0.03)	0.03
QUICKI	0.32 ± 0.03	0.31 ± 0.02	0.31 ± 0.02	0.31 ± 0.02	0.008 (0.001, 0.01)	0.02
Triglycerides (mg/dL)	146.2 ± 67.4	152.4 ± 66.9	139.0 ± 61.3	140.2 ± 64.9	− 8.93 (− 30.54, 12.68)	0.41
VLDL-cholesterol (mg/dL)	29.2 ± 13.5	30.5 ± 13.4	27.8 ± 12.3	28.1 ± 13.0	− 1.78 (− 6.10, 2.53)	0.41
Total cholesterol (mg/dL)	143.5 ± 30.5	146.3 ± 34.0	149.7 ± 26.6	144.6 ± 27.2	− 6.62 (− 18.86, 5.62)	0.28
LDL-cholesterol (mg/dL)	71.2 ± 26.3	73.0 ± 24.1	74.9 ± 22.0	68.1 ± 21.1	− 6.68 (− 15.53, 2.15)	0.13
HDL-cholesterol (mg/dL)	43.0 ± 7.2	42.8 ± 6.2	46.8 ± 6.7	48.4 ± 7.4	2.52 (0.04, 5.00)	0.04
Total-/HDL-cholesterol ratio	3.4 ± 0.8	3.4 ± 0.7	3.2 ± 0.6	3.0 ± 0.6	− 0.27 (− 0.52, − 0.03)	0.02
hs-CRP (mg/L)	4.8 ± 2.5	4.9 ± 2.6	5.1 ± 2.8	4.3 ± 2.6	− 0.88 (− 1.39, − 0.38)	0.001
NO (µmol/L)	46.6 ± 10.0	44.2 ± 8.3	42.4 ± 6.2	46.5 ± 7.4	4.28 (0.66, 7.91)	0.02
TAC (mmol/L)	895.3 ± 301.9	873.3 ± 276.3	965.5 ± 239.4	1044.4 ± 254.9	108.44 (47.61, 169.27)	0.001
GSH (µmol/L)	506.3 ± 96.5	505.3 ± 107.9	586.2 ± 156.3	629.4 ± 169.5	45.15 (5.82, 84.47)	0.02
MDA (µmol/L)	2.7 ± 0.7	2.6 ± 0.4	3.1 ± 1.3	2.7 ± 1.4	− 0.23 (− 0.53, − 0.07)	0.13
SBP (mmHg)	128.3 ± 14.4	127.0 ± 15.1	125.1 ± 12.8	123.2 ± 13.3	− 1.30 (− 5.96, 3.35)	0.57
DBP (mmHg)	79.3 ± 8.8	78.2 ± 8.5	77.9 ± 6.9	76.2 ± 8.0	− 1.08 (− 3.51, 1.35)	0.37

Data are mean ± SDs

*DBP* diastolic blood pressure, *FPG* fasting plasma glucose, *GSH* total glutathione, *HOMA-IR* homeostasis model of assessment-estimated insulin resistance, *hs-CRP* high-sensitivity C-reactive protein, *MDA* malondialdehyde, *NO* nitric oxide, *QUICKI* quantitative insulin sensitivity check index, *SBP* systolic blood pressure, *TAC* total antioxidant capacity

^a^”Outcome measures” refers to the change in values of measures of interest between baseline and week 12. β [difference in the mean outcomes measures between treatment groups (probiotic group = 1 and placebo group = 0)]

^2^Obtained from multiple regression model (adjusted for baseline values of each biochemical variables, age and baseline BMI)

## Discussion

This study demonstrated that taking probiotics for 12 weeks by diabetic patients with CHD had beneficial effects on glycemic control, improving HDL levels- and total-/HDL-cholesterol ratio, and attenuating biomarkers of inflammation and oxidative stress. The observed difference in glycemic control, HDL-, total-/HDL-cholesterol ratio, inflammatory markers and oxidative stress in our study was statistically significant between two intervention groups, however, it was not clinically significant. Long-term interventions and higher dosages of probiotic supplements might result in greater changes in these metabolic profiles.

### Effects on glycemic control and lipid profiles

A few studies have evaluated the effects of probiotic supplementation on glycemic control and lipid profiles among diabetic patients; however this data is scarce among diabetic patients with CHD. In a meta-analysis conducted by Yao et al. [22], probiotic ingestion significantly reduced HbA1c and insulin levels in patients with T2DM, but did not affect their lipid profiles. In addition, Taylor et al. [7]. found that probiotic supplementation for 6–8 weeks resulted in a significant reduction in insulin resistance in pregnant women with GDM, but did not influence their lipid profiles and fasting glucose levels. Furthermore, it was reported that gut microbiota contributes to glucose homeostasis through different bacterial metabolites [23]. Previous meta-analyses revealed that probiotic supplementation decreased insulin resistance and glycated hemoglobin levels [24, 25]. In contrast, such beneficial effects were not reported by others [26–29]. Insulin resistance and dyslipidemia in patients with T2DM increase the risk of microvascular complications and cardiovascular mortality [30]. Therefore, probiotic administration might be useful to decrease diabetic complications, due to its glucose-lowering effect. Improved glucose homeostasis parameters, HDL- and total-/HDL-cholesterol ratio by probiotics among diabetic patients with CHD may be related to their role in decreasing inflammatory cytokines and suppressing the nuclear factor-κB pathway [31], their impact on gene expression [12] and the activation of gut microbiota-short chain fatty acids (SCFA)-hormone axis [32].

### Effects on biomarkers of inflammation and oxidative stress

We have previously shown that taking probiotics by patients with major depressive disorder for 8 weeks improved hs-CRP and GSH concentrations, but did not influence TAC levels [28]. Furthermore, a significant reduction in hs-CRP levels was seen after taking probiotic supplements for 48 weeks by HIV-infected individuals [33]. On the other hand, no significant changes in MDA and TAC levels were found following probiotic capsule supplementation containing *Lactobacillus casei* (10^8^ CFU/g) for 8 weeks in patients with rheumatoid arthritis [34]. Diabetes is correlated with enhanced concentrations of vascular inflammation and oxidative stress parameters [35]. Cell culture studies suggest that elevated pro-inflammatory markers may cause a reduction in GSH synthesis [36, 37]. Discrepancies in these findings may be explained through the differences in study design, characteristics of study populations, probiotic species, strains and formulations available, and duration of the intervention. Probiotics ingestion may decrease inflammation and oxidative stress through increased production of SCFA in the colon, improved activity of glutamate-cysteine-ligase activity (GCL) and increased gene expression of GCL subunits [38].

This study had few limitations. In the current study, fecal bacterial loads were not measured before and after probiotic consumption. Also, there was no chance to evaluate gene expression of lipid, insulin, inflammation and oxidative damage in diabetic patients with CHD.

## Conclusions

Overall, probiotic supplementation for 12 weeks had beneficial effects on glycemic control, HDL-cholesterol, total-/HDL-cholesterol ratio, biomarkers of inflammation and oxidative stress in diabetic patients with CHD. Our findings clarify that probiotic supplementation may confer advantageous therapeutic impacts for diabetic patients with CHD. Further research is required in other populations and for longer period of time to determine the beneficial effects of probiotic supplementation.

## Abbreviations

FPG: fasting plasma glucose
GSH: total glutathione
HOMA-IR: homeostasis model of assessment-insulin resistance
HDL-cholesterol: high density lipoprotein-cholesterol
Hs-CRP: high sensitivity C-reactive protein
LDL-cholesterol: low density lipoprotein-cholesterol
MDA: malondialdehyde
NO: nitric oxide
QUICKI: quantitative insulin sensitivity check index
VLDL-cholesterol: very low density lipoprotein-cholesterol
TAC: total antioxidant capacity
